# Comparative diagnostic performance of C-TIRADS versus Kwak TI-RADS for thyroid nodules: implications for fine-needle aspiration biopsy referral

**DOI:** 10.3389/fendo.2026.1743112

**Published:** 2026-06-04

**Authors:** Li Xia, Anjie Chen, Li Kang, Shiming Guan, Qing Lyu

**Affiliations:** 1 Department of General Surgery, Jiangnan University Medical Center, Wuxi, Jiangsu, China; 2 Wuxi School of Medicine, Jiangnan University, Wuxi, Jiangsu, China; 3 Department of Ultrasound Medicine, Affiliated Hospital of Jiangnan University, Wuxi, Jiangsu, China; 4 Department of Geriatric Medicine, Shanghai Health And Medical Center (Huadong Sanatorium), Wuxi, Jiangsu, China; 5 Department of Thyroid and Breast Surgery, Affiliated Hospital of Jiangnan University, Wuxi, Jiangsu, China

**Keywords:** C-TIRADS, diagnostic performance, Kwak TI-RADS, thyroid nodule, ultrasonographic features

## Abstract

**Background:**

Accurate differentiation between malignant and benign thyroid nodules is essential. Although ultrasound serves as the primary diagnostic modality, there is a paucity of longitudinal data concerning nodule progression and limited validation of the Chinese Thyroid Imaging Reporting and Data System (C-TIRADS). This underscores the necessity for evidence-based follow-up strategies. The present study sought to assess the developmental trends of thyroid nodules over an extended follow-up period and to evaluate the diagnostic efficacy of the C-TIRADS.

**Methods:**

This was a single-center retrospective longitudinal cohort study. We included patients who underwent thyroid ultrasound between 2019 and 2024 with at least one follow-up examination. Exclusions were: (1) prior thyroid malignancy; (2) incomplete baseline data; (3) follow-up <12 months without pathology/cytology. In the primary analysis, the nodule was the statistical unit. Correlation among multiple nodules within the same patient was addressed by clustering at the patient level. A sensitivity analysis at the patient level retained only the largest or most suspicious nodule per patient. Two radiologists (≥5 years’ experience) independently graded nodules using C-TIRADS and Kwak Thyroid Imaging Reporting and Data System (Kwak TI-RADS) while blinded to the reference standard. Disagreements were adjudicated by a senior radiologist. Inter-reader agreement was reported using the weighted kappa (κ). The reference standard was surgical histopathology whenever available. For non-surgical cases, cytology Bethesda V/VI was considered malignant; nodules with ≥24 months of stable imaging (no upgrade or suspicious progression) were considered benign. Indeterminate cases were excluded from the primary endpoint analysis. We reported the verification proportion and performed two sensitivity analyses: (1) surgery-only subset; (2) including nodules deemed benign after ≥24-month stable follow-up. For predefined thresholds (e.g., C-TIRADS ≥4B, Kwak ≥4), 2×2 tables were used to derive sensitivity, specificity, positive predictive value (PPV), and negative predictive value (NPV) with 95% CIs. Area Under the Curves (AUCs) were estimated and compared using the DeLong method. Because nodules were clustered within patients, Generalized Estimating Equations (GEE) with an exchangeable correlation structure or cluster-robust standard errors were applied. Temporal changes in sonographic features were assessed with trend tests. Missing data were handled by complete-case analysis. Ultrasonographic data from patients diagnosed with thyroid nodules in 2019 and monitored consistently until 2024 were analyzed to evaluate longitudinal changes in nodule characteristics. A retrospective analysis of 272 surgically confirmed cases (173 malignant and 99 benign) was performed to compare the diagnostic efficacy of the C-TIRADS with the Kwak TI-RADS.

**Results:**

1.Throughout the follow-up period, the nodules identified in the study participants were predominantly multiple and bilaterally distributed across the thyroid lobes, with their prevalence increasing annually. The majority of these nodules were round or oval in shape, with diameters less than 10 mm. However, there was a gradual increase in the proportion of nodules exceeding 10 mm in diameter, as well as those that were oval or irregularly shaped. Ultrasound characteristics such as microcalcifications, border invasion, a taller-than-wide shape (aspect ratio ≥1), and internal vascularity were largely absent in most nodules. Nonetheless, there was an upward trend in the proportion of nodules exhibiting these features. Nodules characterized by internal hypoechogenicity and lacking posterior acoustic attenuation constituted a significant proportion and did not demonstrate a notable trend of change over time. 2. The AUC for the C-TIRADS classification in the diagnosis of nodules was determined to be 0.784. A C-TIRADS category of ≥4B was identified as the optimal cutoff value for the diagnosis of malignant nodules, yielding sensitivity, specificity, PPV, NPV, and accuracy rates of 75.72%, 75.76%, 84.52%, 64.10%, and 75.74%, respectively. Furthermore, no statistically significant difference (P > 0.05) was observed in the diagnostic efficacy between the C-TIRADS classification and the Kwak TI-RADS classification for thyroid nodules.

**Conclusion:**

Microcalcifications, irregular margins, a taller-than-wide morphology, and internal vascularity were increasingly observed during follow-up and may be associated with malignancy, warranting closer surveillance. The C-TIRADS demonstrates diagnostic accuracy comparable to that of the Kwak TI-RADS, thereby reinforcing its clinical utility in the management of thyroid nodules.

## 1 Introduction

Thyroid nodules represent one of the most prevalent disorders within the endocrine system. Numerous studies have reported that the prevalence of thyroid nodules in China ranges from 20% to 40%, with a higher incidence observed in females (approximately 34% to 40%) compared to males (approximately 20% to 32%). Thyroid nodules are classified into benign and malignant categories, with malignant nodules comprising 5% to 15% of cases (1–6). Malignant nodules frequently necessitate surgical intervention (7), whereas benign nodules generally require only regular follow-up. Consequently, accurately assessing the nature of thyroid nodules is essential for determining appropriate clinical management strategies. Diagnostic techniques for thyroid nodules include palpation, ultrasound, and ultrasound-guided fine-needle aspiration biopsy (FNAB) (8). Among these, ultrasound is the preferred diagnostic modality due to its simplicity, cost-effectiveness, absence of radiation, high resolution, and superior detection rate for thyroid nodules. In addition, ultrasound is recognized as the most valuable imaging modality for the follow-up monitoring of thyroid nodules (9), facilitating the detection of nodule progression or changes, thus informing optimal treatment strategies and reducing healthcare expenditures. Ultrasound-based risk stratification systems play a central role in the management of thyroid nodules. Among them, Kwak TI-RADS is one of the most widely recognized and frequently adopted classification systems and has served as an important reference framework in previous thyroid nodule studies. In contrast, the Chinese Thyroid Imaging Reporting and Data System (C-TIRADS) was proposed to better fit the Chinese clinical setting and was incorporated into the 2020 Chinese guidelines for ultrasound malignancy risk stratification of thyroid nodules (10). Therefore, comparing C-TIRADS with Kwak TI-RADS is clinically meaningful for evaluating whether C-TIRADS provides comparable diagnostic performance while being more applicable to domestic practice. This study examined thyroid ultrasound findings from individuals diagnosed with thyroid nodules in 2019 at Huadong Sanatorium or the Affiliated Hospital of Jiangnan University, who underwent regular follow-up until 2024. The aim was to investigate the ultrasonographic characteristics and longitudinal evolution of thyroid nodules in a long-term follow-up cohort. Moreover, a retrospective analysis was conducted on ultrasound results from 272 patients who exhibited suspicious malignant features during follow-up and subsequently underwent thyroid nodule surgery. The diagnostic performance of the Chinese Thyroid Imaging Reporting and Data System (C-TIRADS) for thyroid nodules was evaluated and compared with the widely utilized Kwak Thyroid Imaging Reporting and Data System (Kwak TIRADS) classification criteria. Accordingly, the primary objective of this study was to determine whether C-TIRADS provides diagnostic performance comparable to that of Kwak TI-RADS for differentiating malignant from benign thyroid nodules, while also evaluating its potential utility in longitudinal surveillance and FNAB referral in routine clinical practice.

## 2 Methods

### 2.1 Study design and population

This was a retrospective longitudinal cohort study conducted at Huadong Sanatorium and the Affiliated Hospital of Jiangnan University. This retrospective study was conducted in accordance with the Declaration of Helsinki and was approved by the Ethics Committee of the Affiliated Hospital of Jiangnan University (Approval No. LS2025170). The requirement for informed consent was waived by the same committee due to the retrospective nature of the study and the use of anonymized patient data. Patients with thyroid nodules detected by ultrasound in 2019 and followed through 2024 were eligible for inclusion.

Inclusion criteria:

Participants included those with thyroid nodules identified via ultrasound in 2019, who also completed annual or biennial physical examinations with comprehensive ultrasound documentation at the aforementioned institutions from the point of nodule detection through to 2024.

Exclusion Criteria: Individuals under the age of 18, those with a history of long-term use of immunosuppressants, hormonal therapies, or iodine-containing medications, and those with missing or incomplete ultrasound records were excluded.

During the follow-up period, 272 patients presenting with ultrasound features suggestive of malignancy underwent surgical intervention for thyroid nodules. These suspicious features mainly included microcalcifications, irregular or ill-defined/spiculated margins, taller-than-wide shape, marked hypoechogenicity, and other sonographic findings concerning for malignancy in routine clinical assessment. Surgical decisions were based on comprehensive clinical judgment rather than a single predefined study criterion. Pathological analysis confirmed malignancy in 173 cases, while 99 cases were benign. The ultrasound data and pathological findings from these patients were employed to assess the diagnostic efficacy of the C-TIRADS classification system for thyroid nodules.

### 2.2 Research methods

All participants enrolled in the study underwent thyroid ultrasound examinations utilizing a GE E9 color Doppler ultrasound system equipped with a transducer frequency range of 5–13 MHz. Comprehensive demographic data, including name, sex, and age, along with ultrasonographic parameters, were systematically collected. The ultrasound dataset encompassed various nodule characteristics: the number of nodules, size (measured by maximum diameter), distribution (unilateral or bilateral), internal echogenicity (categorized as very hypoechoic, mildly hypoechoic, isoechoic, hyperechoic, or mixed echogenicity), and posterior acoustic features (presence or absence of attenuation). Although posterior acoustic features may include enhancement, shadowing, no posterior feature, or combined patterns, these detailed subtypes were not consistently available in all retrospective records and were therefore summarized based on available data. Additionally, morphological features were assessed, including shape (round, oval, or irregular), presence of microcalcifications, border invasion (characterized by ill-defined margins, spiculated margins, or extrathyroidal extension), taller-than-wide ratio(≥1), and intranodular vascularity evaluated through Doppler imaging. Similarly, vascular patterns may be classified into multiple subtypes according to peripheral and central flow combinations; however, due to limitations of retrospective data collection, detailed subclassification was not uniformly available and thus simplified in the present analysis. For those patients who underwent surgical intervention (n=272), thyroid nodule ultrasound images were retrospectively analyzed using both the C-TIRADS and Kwak TI-RADS classification systems. These systems assessed critical indicators of malignancy to categorize nodules into risk categories.

### 2.3 Statistical analyses

The data analysis was conducted utilizing SPSS version 26.0 and MedCalc version 20.0 statistical software. The relationship between the prevalence of ultrasonographic nodule features and the duration of follow-up was assessed using the Chi-square test, with statistical significance established at P < 0.05. Employing histopathological outcomes as the reference standard, the sensitivity, specificity, positive predictive value (PPV), negative predictive value (NPV), and accuracy of the C-TIRADS and Kwak TI-RADS classifications were determined. Receiver operating characteristic curves (ROC) were generated to ascertain the AUC and to identify optimal cutoff values for malignancy prediction. Comparisons of AUCs between the two classification systems were conducted using the Z-test. Variations in sensitivity, specificity, PPV, NPV, and accuracy were evaluated using the paired Chi-square test (McNemar’s test for paired proportions). Statistical significance was defined as P < 0.05 for all analyses.

## 3 Results

### 3.1 Longitudinal changes in ultrasonographic features from 2019 to 2024

The year-by-year distribution of ultrasonographic features of thyroid nodules is summarized in **Table 1**. Throughout the follow-up period, multiple nodules were more common than solitary nodules, and the proportion of multiple nodules increased steadily from 57.41% in 2019 to 75.94% in 2024 (χ² = 830.03, P < 0.05). Most nodules remained smaller than 10 mm; however, the proportions of medium-sized (10–20 mm) and large (>20 mm) nodules gradually increased over time (χ² = 215.03, P < 0.05).

**Table 1:** Year-by-year distribution of ultrasonographic features of thyroid nodules, 2019-2024, n/N (%).

Ultrasound Sign		2019	2020	2021	2022	2023	2024	χ^2^	*P*
Number of nodules	Single	3692(42.59)	2627(36.39)	2394(31.84)	1549(29.83)	1781(25.56)	1523(24.06)	830.03	<0.05
	Multiple	4976(57.41)	4593(63.61)	5124(68.16)	3644(70.17)	5186(74.44)	4806(75.94)		
Diameter of nodules	Small	7119 (82.13)	5792 (80.22)	5890 (78.35)	4060 (78.18)	5249 (75.34)	4665 (73.71)	215.03	<0.05
	Medium	1346(15.53)	1229(17.02)	1412(18.78)	977 (18.81)	1457(20.91)	1394(22.03)		
	Large	203 (2.34)	199 (2.76)	216 (2.87)	156 (3.00)	261 (3.75)	270 (4.27)		
Distribution of nodules	Left lobe	2255(26.02)	1671(23.14)	1606(21.36)	1040(20.03)	1267(18.19)	1109(17.52)	676.06	<0.05
	Right lobe	2500(28.84)	1883(26.08)	1794(23.86)	1221(23.51)	1425(20.45)	1249 (19.73)		
	Bilateral lobe	3848(44.39)	3591(49.74)	4022(53.50)	2860(55.07)	4170 (59.85)	3857 (60.94)		
	Lateral lobe + isthmus / isthmus	65 (0.75)	75 (1.04)	96 (1.28)	72 (1.39)	105 (1.51)	114 (1.80)		
Shape of nodules	Round	7581(87.46)	6099(84.47)	6048(80.45)	3617(69.65)	3752(53.85)	2763(43.66)	5445.73	<0.05
	Oval	1056(12.18)	1088(15.07)	1436(19.10)	1551(29.87)	3171(45.51)	3501(55.32)		
	Irregular	31 (0.36)	33 (0.46)	34 (0.45)	25 (0.48)	44 (0.63)	65 (1.03)		
Nodal punctate echogenic foci	Yes	226 (2.61)	239 (3.31)	264 (3.51)	192 (3.70)	253 (3.63)	243 (3.84)	23.38	<0.05
	No	8442(97.39)	6981(96.69)	7254(96.49)	5001(96.30)	6714(96.37)	6086(96.16)		
Nodal irregular/ill-defined margins	Yes	143 (1.65)	157 (2.17)	160 (2.13)	147 (2.83)	183 (2.63)	187 (2.95)	38.97	<0.05
	No	8525(98.35)	7063(97.83)	7358(97.87)	5046(97.17)	6784(97.37)	6142(97.05)		
Nodal aspect ratio	≥1	28 (0.32)	41 (0.57)	48 (0.64)	34 (0.65)	52 (0.75)	45 (0.71)	15.70	<0.05
	<1	8640(99.68)	7179(99.43)	7470(99.36)	5159(99.35)	6915(99.25)	6284(99.29)		
Blood flow detected in nodules	Yes	110 (1.27)	116 (1.61)	139 (1.85)	114 (2.20)	138 (1.98)	158 (2.50)	37.10	<0.05
	No	8558(98.73)	7104(98.39)	7379(98.15)	5079(97.80)	6829(98.02)	6171(97.50)		
Internal echogenicity of the nodules	Very low echo	50 (0.58)	50 (0.69)	50 (0.67)	50 (0.96)	50 (0.72)	50 (0.79)	16.8	0.67
	Slightly low echo	7861(90.69)	6495(89.96)	6750(89.78)	4687(90.26)	6271(90.01)	5726(90.47)		
	Equal Echo	145 (1.67)	141 (1.95)	137 (1.82)	85 (1.64)	129 (1.85)	115 (1.82)		
	High Echo	98 (1.13)	88 (1.22)	89 (1.18)	67 (1.29)	85 (1.22)	78 (1.23)		
	Mixed Echo	514 (5.93)	446 (6.18)	492 (6.54)	304 (5.85)	432 (6.20)	360 (5.69)		
Posterior echoes of the nodules	With attenuation	40 (0.46)	36 (0.50)	36 (0.48)	22 (0.42)	34 (0.49)	30 (0.47)	0.43	0.994
	No attenuation	8628(99.54)	7184(99.50)	7482(99.52)	5171(99.58)	6933(99.51)	6299(99.53)		

With regard to nodule distribution, bilateral involvement became increasingly common during follow-up, rising from 44.39% in 2019 to 60.94% in 2024, whereas unilateral nodules became less frequent (χ² = 676.06, P < 0.05). In terms of morphology, the proportion of round nodules decreased over time, while oval- and irregular-shaped nodules increased significantly (χ² = 5445.73, P < 0.05).

Several suspicious sonographic features also became more prevalent during follow-up. The proportion of nodules with punctate echogenic foci (microcalcifications) increased from 2.61% to 3.84% (χ² = 23.38, P < 0.05), irregular or ill-defined margins from 1.65% to 2.95% (χ² = 38.97, P < 0.05), a taller-than-wide shape from 0.32% to 0.71% (χ² = 15.70, P < 0.05), and intranodular vascularity from 1.27% to 2.50% (χ² = 37.10, P < 0.05).

By contrast, internal echogenicity showed no significant change over time (χ² = 16.8, P = 0.67), and posterior acoustic features also remained stable throughout follow-up (χ² = 0.43, P = 0.994).

### 3.2 Diagnostic performance of C-TIRADS and Kwak TI-RADS

A total of 272 surgically confirmed thyroid nodules were included in the diagnostic performance analysis, including 173 malignant nodules and 99 benign nodules. The diagnostic indices of C-TIRADS and Kwak TI-RADS at different thresholds are shown in **Table 2**.

**Table 2:** Diagnostic performance of C-TIRADS and Kwak TI-RADS at prespecified thresholds in the long-term follow-up cohort.

System	Threshold	Sensitivity (%)	Specificity (%)	PPV (%)	NPV (%)	Accuracy (%)
Kwak TI-RADS	≥4A	91.91	25.25	68.24	64.10	67.65
Kwak TI-RADS	≥4B	73.99	77.78	85.33	63.11	75.37
Kwak TI-RADS	≥4C	37.57	94.95	92.86	46.53	58.46
C-TIRADS	≥4A	92.49	24.24	68.09	64.86	67.65
C-TIRADS	≥4B	75.72	75.76	84.52	64.10	75.74
C-TIRADS	≥4C	41.62	92.93	91.14	47.67	60.29

PPV, positive predictive value; NPV, negative predictive value.

For Kwak TI-RADS, the threshold of ≥4A yielded high sensitivity (91.91%) but low specificity (25.25%), whereas ≥4C yielded high specificity (94.95%) but low sensitivity (37.57%). The threshold of ≥4B provided the most balanced diagnostic performance, with a sensitivity of 73.99%, specificity of 77.78%, PPV of 85.33%, NPV of 63.11%, and accuracy of 75.37%.

Similarly, for C-TIRADS, the threshold of ≥4A showed high sensitivity (92.49%) but low specificity (24.24%), while ≥4C showed high specificity (92.93%) but relatively low sensitivity (41.62%). Among the evaluated thresholds, C-TIRADS ≥4B demonstrated the best balance between sensitivity and specificity, with a sensitivity of 75.72%, specificity of 75.76%, PPV of 84.52%, NPV of 64.10%, and accuracy of 75.74%.

**Figure 1:**
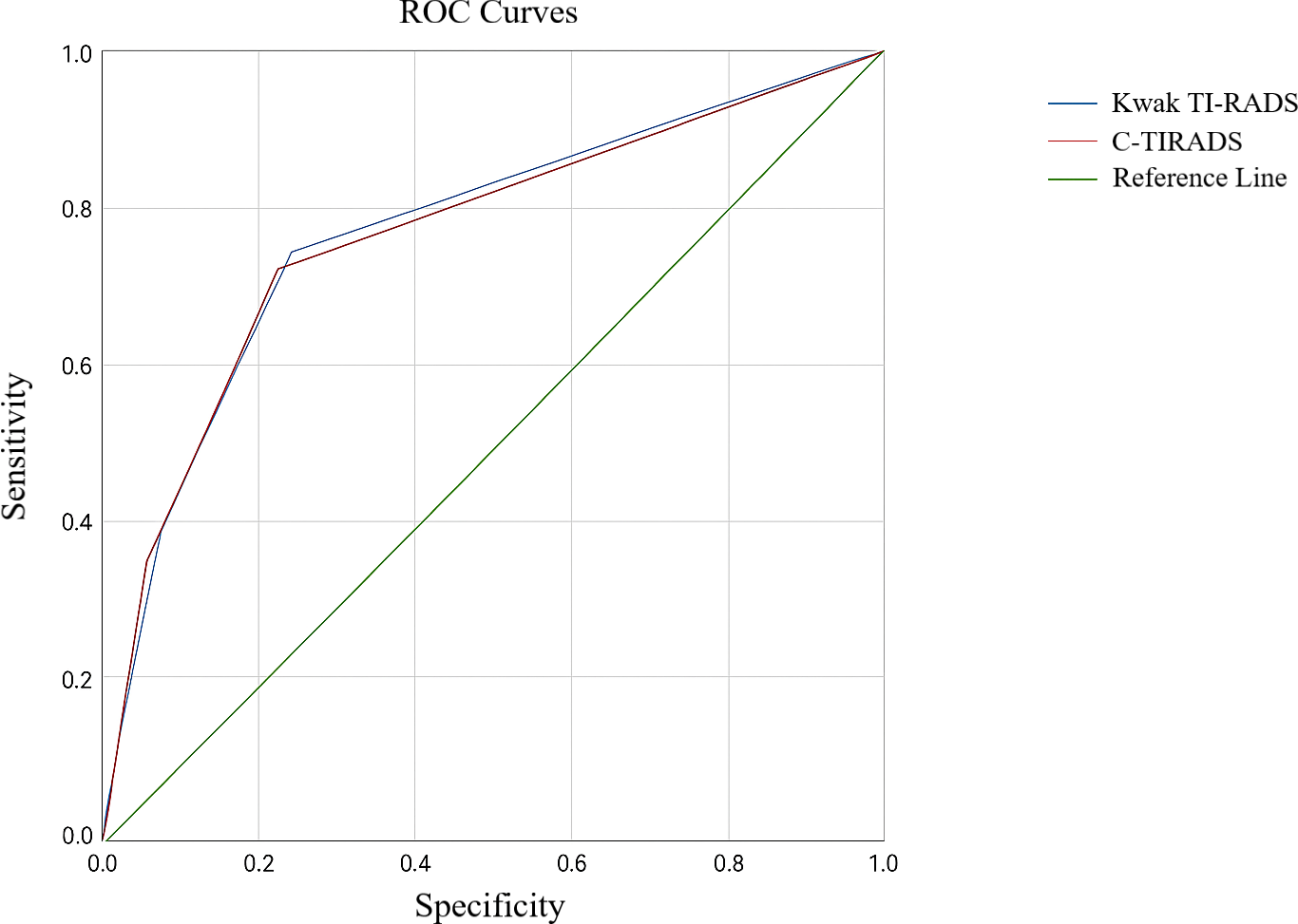
The areas under the ROC curve for diagnosing malignant thyroid nodules were 0.784 (95% CI: 0.729–0.840) for the C-TIRADS classification and 0.785 (95%CI: 0.730–0.840)​for the Kwak TI-RADS classification, with no statistically significant difference between them.

Receiver operating characteristic analysis showed that the AUC was 0.784 (95% CI: 0.729–0.840) for C-TIRADS and 0.785 (95% CI: 0.730–0.840) for Kwak TI-RADS (**Figure 1**). There was no statistically significant difference in AUC between the two classification systems (P > 0.05).

## 4 Discussion

A longitudinal examination of ultrasonographic characteristics in patients with thyroid nodules over an extended follow-up period yields essential insights into the dynamic progression of these nodules. This understanding aids in the formulation of standardized and rational follow-up strategies that are customized to individual clinical profiles. Additionally, the incorporation of the C-TIRADS classification or other validated systems for evaluating nodule characteristics facilitates a straightforward and cost-effective distinction between benign and malignant lesions. This approach enables the timely identification of malignant transformations and the initiation of suitable therapeutic interventions (4, 11).

Existing research indicates that characteristics such as nodule number, size, distribution, and intranodular vascularity currently lack sufficient specificity to independently predict malignancy. However, these features may influence follow-up protocols (12–14). In this study, the observed increase in the prevalence of multiple nodules, medium to large-sized nodules, bilateral or isthmus-involving nodules, and nodules with detectable vascularity over time suggests that thyroid nodules tend to grow in size and number with advancing age. Nevertheless, these changes should not be construed as indicative of malignant potential. Based on the observed trends, interval changes in nodule morphology should receive closer attention during follow-up. Nodules exhibiting rapid growth should prompt shortened follow-up intervals and consideration of FNAB when clinically warranted.

Furthermore, it is imperative to advise patients against excessive anxiety concerning increases in the number, size, or vascularity of nodules. Clinical attention should be directed towards identifying high-risk sonographic markers, such as microcalcifications, irregular margins, and nonparallel orientation (15, 16). This approach should be complemented by optimizing follow-up schedules and tailoring diagnostic and therapeutic strategies based on individualized risk stratification.

Ultrasonographic characteristics, such as nodule shape, border integrity, microcalcifications, and the taller-than-wide ratio (aspect ratio ≥1), are widely acknowledged as key indicators for evaluating malignancy risk in thyroid nodules and are integrated into most classification systems, including C-TIRADS. However, research indicates that while microcalcifications and a taller-than-wide ratio exhibit high specificity for malignancy, their sensitivity is limited (17, 18). Notably, the sensitivity of the taller-than-wide ratio decreases progressively with increasing nodule size. In this cohort, the majority of nodules presented with round or oval shapes, well-defined borders, aspect ratios <1, and lacked microcalcifications (19). Nonetheless, the proportion of nodules with irregular shapes, ill-defined or spiculated borders, microcalcifications, or aspect ratios ≥1 increased during follow-up, suggesting potential malignant transformation in a subset of cases. These findings highlight the importance of prioritizing shape, border integrity, microcalcifications, and aspect ratio in long-term follow-up protocols (20–24).

To address diagnostic limitations, it is essential to enhance sensitivity for nodules with an aspect ratio of ≥1. As nodule enlargement during follow-up can diminish the diagnostic sensitivity of a taller-than-wide ratio, employing multi-planar ultrasonographic imaging, such as transverse and longitudinal views, is recommended to improve detection accuracy in larger nodules (25–28). Furthermore, optimizing the detection of microcalcifications is crucial, given the low sensitivity associated with their identification. This necessitates the use of meticulous scanning techniques and high-resolution ultrasound equipment to reduce the likelihood of overlooking small calcific foci. Clinically, it is imperative for radiologists to undergo targeted training to identify subtle malignant features in evolving nodules. Institutions should also consider upgrading to high-frequency transducers, such as those in the range of 15–18 MHz, to enhance resolution in follow-up examinations (19, 29).

Marked hypoechogenicity and posterior acoustic attenuation are recognized as potential indicators of malignancy, hypothesized to result from densely packed and overlapping cancer cells (30). However, in this study, neither marked hypoechogenicity nor posterior acoustic attenuation demonstrated significant trends of change during follow-up. Similarly, the proportions of nodules with mild hypoechogenicity, isoechogenicity, hyperechogenicity, or mixed echogenicity remained stable over time. Furthermore, within the surgical cohort, very few cases exhibited these features, thereby excluding the confounding effect of surgical selection on prevalence trends. Potential explanations for these findings include the inherent stability of echogenic patterns: the internal and posterior acoustic characteristics of thyroid nodules may be established during their initial formation and remain largely unaffected by factors such as disease duration, nodule size, or vascularity (31). Reevaluation of Existing Malignancy Theory: The prevailing hypothesis that malignant transformation leads to changes in echogenicity through the disruption of cellular architecture may necessitate revision. In this cohort, the transition from benign to malignant states did not correspond with changes in echogenic characteristics. Consequently, we suggest that significant hypoechogenicity and posterior acoustic attenuation should be prioritized solely during the initial ultrasound assessment. In follow-up examinations, these features should be emphasized only if newly observed, thereby enhancing the efficiency of resource utilization in long-term monitoring (32).

In 2020, Zhan et al. introduced the C-TIRADS, a classification framework specifically designed to address the clinical and healthcare context of China (33). Notable advancements of C-TIRADS over existing systems such as Kwak TI-RADS include the substitution of hypoechogenicity with marked hypoechogenicity as a malignancy risk criterion, and the incorporation of comet-tail artifacts (indicative of benignity) as a negative scoring element to enhance risk stratification. Utilizing surgical histopathology as the reference standard, C-TIRADS demonstrated diagnostic performance comparable to that of Kwak TI-RADS, with the C-TIRADS category 4B showing malignancy prediction efficacy akin to Kwak TI-RADS 4B. The development of C-TIRADS is closely aligned with the healthcare landscape in China, where the adoption of FNAB for thyroid nodules is limited, indicating its practical applicability and potential for widespread implementation in domestic clinical practice (34). Nevertheless, the influence of marked hypoechogenicity and comet-tail artifacts on the diagnostic accuracy of C-TIRADS has yet to be comprehensively elucidated. Future research should aim to validate these characteristics across diverse cohorts and enhance their integration into risk assessment protocols (35, 36).

The limitations of this study are as follows: In the examination of ultrasonographic features of thyroid nodules during follow-up, certain imaging characteristics, including internal structure and sonographic halo, were not analyzed due to incomplete data collection, leading to a lack of comprehensive coverage of all pertinent features. Another limitation is that this was a retrospective study based on routine ultrasound records. Some detailed subclassifications defined in C-TIRADS, including posterior acoustic features and vascular patterns, were not consistently available and therefore could not be fully analyzed. The assessment of the diagnostic efficacy of the C-TIRADS classification was conducted using a retrospective study design, which inherently possesses lower data quality compared to prospective studies. Additionally, the relatively high prevalence of malignant nodules within the study population may have introduced bias into the research findings.

In conclusion, during the long-term monitoring of thyroid nodules, it is imperative to closely monitor the morphological characteristics, including shape, margin, microcalcifications, and aspect ratio. Proactive strategies should be employed to enhance diagnostic sensitivity. Internal echogenicity and posterior acoustic features require thorough assessment only during the initial evaluation and do not demand specific attention in subsequent follow-ups. The C-TIRADS classification exhibits satisfactory diagnostic performance for thyroid nodules, with C-TIRADS ≥4B serving as the optimal threshold for malignancy diagnosis. The diagnostic efficacy of C-TIRADS is comparable to that of the Kwak TI-RADS classification. Nonetheless, the clinical significance of extremely hypoechoic features and comet-tail artifacts within the C-TIRADS framework requires further exploration.

## 5 Conclusions

In conclusion, during long-term follow-up of thyroid nodules, particular attention should be paid to changes in shape, margins, microcalcifications, taller-than-wide orientation, and vascularity, as these features may indicate increased malignancy risk. By contrast, internal echogenicity and posterior acoustic features did not show significant temporal variation in this cohort. C-TIRADS demonstrated diagnostic performance comparable to that of Kwak TI-RADS, and C-TIRADS ≥4B appeared to be the most appropriate threshold for malignancy prediction in this cohort, supporting its clinical usefulness in thyroid nodule risk stratification and FNAB referral.

## Data Availability

The original contributions presented in the study are included in the article/supplementary material. Further inquiries can be directed to the corresponding authors.
